# Ambiguity Resolution for Phase-Based 3-D Source Localization under Fixed Uniform Circular Array

**DOI:** 10.3390/s17051086

**Published:** 2017-05-11

**Authors:** Xin Chen, Zhen Liu, Xizhang Wei

**Affiliations:** School of Electronic Science and Engineering, National University of Defense Technology, Changsha 410073, China; chenxin10@nudt.edu.cn (X.C.); liweier@nudt.edu.cn (X.W.)

**Keywords:** 3-D source localization, phase ambiguity, uniform circular array, subarray grouping, ambiguity searching

## Abstract

Under fixed uniform circular array (UCA), 3-D parameter estimation of a source whose half-wavelength is smaller than the array aperture would suffer from a serious phase ambiguity problem, which also appears in a recently proposed phase-based algorithm. In this paper, by using the centro-symmetry of UCA with an even number of sensors, the source’s angles and range can be decoupled and a novel algorithm named subarray grouping and ambiguity searching (SGAS) is addressed to resolve angle ambiguity. In the SGAS algorithm, each subarray formed by two couples of centro-symmetry sensors can obtain a batch of results under different ambiguities, and by searching the nearest value among subarrays, which is always corresponding to correct ambiguity, rough angle estimation with no ambiguity is realized. Then, the unambiguous angles are employed to resolve phase ambiguity in a phase-based 3-D parameter estimation algorithm, and the source’s range, as well as more precise angles, can be achieved. Moreover, to improve the practical performance of SGAS, the optimal structure of subarrays and subarray selection criteria are further investigated. Simulation results demonstrate the satisfying performance of the proposed method in 3-D source localization.

## 1. Introduction

Passive source localization using an array of sensors plays an increasingly important role in wireless communication, electronic reconnaissance, sonar and other applications [1,2,3,4]. As uniform circular array (UCA) has several advantages over the other array geometries, such as its 360° azimuth angle coverage, almost identical beamwidth and additional elevation angle information [5,6], it is widely applied in three-dimensional (3-D) source localization.

Currently, in the context of 3-D parameter estimation, many effective solutions mainly focus on two types, which are spectrum-based and phase-based estimators [7,8,9]. The spectrum-based estimators, such as multiple signal classification (MUSIC) algorithms [8,10], can achieve source localization with high precision, but suffer from a high computational cost. To cope with this problem, a series of phase-based solutions have been recently proposed in [11,12], which are computationally simpler, since multidimensional search is not required. Although the estimation performance of these approaches is acceptable, for fixed UCA, in practice, when the array aperture is larger than source’s half-wavelength they would suffer from a serious phase ambiguity problem, which will lead to inaccuracies in 3-D parameter estimation.

In the case of ambiguity resolution under UCA, by utilizing the nonlinear property of pseudo direction of arrival (DOA) estimation, a rotary way to resolve ambiguity for the 3D-MUSIC algorithm was proposed in [13], which can suppress pseudo peaks of spectrum and obtain the actual source’s parameters. Referring to the idea of ambiguity resolution via rotating interferometer [14,15], we have recently proposed an improved, unambiguous phase-based algorithm in [16], which performs adaptive rotation to make the sensors form a virtual short baseline. Even so, before and after rotation, as the location of moving sources varies or the two groups of receiving data of multiple frequency-hopping sources can not correspond to the same source, these rotary ways will be inapplicable.

In this paper, without rotation, we address unambiguous 3-D source localization by utilizing different ambiguity properties of each subarray divided by a fixed UCA, and each subarray can obtain the actual source’s parameters under a certain ambiguity. A novel method called subarray grouping and ambiguity searching (SGAS) will be proposed. In SGAS, we first note that the ambiguity is mainly induced by angle parameters, and that the separation of the source’s angles and range is necessary as both are interlaced with each other, which causes the difficulty of ambiguity resolution. To resolve this trouble, we refer to [9,12] and find that the source’s range can be counteracted by computing the phase differences of receiving data from centro-symmetric sensors (CSS), thus source’s angles and range could be decoupled. Then, based on the traverse of ambiguity and some algebraic operations, each subarray with the same structure formed by two couples of CCS can obtain a batch of results under different ambiguities, and the rough angle estimation can be achieved by searching the nearest value among subarrays. Finally, to realize source’s range as well as more precise angle estimation, the unambiguous angles obtained by SGAS are utilized to resolve ambiguity in the phase-based algorithm [11] via the approximation of steering vectors between planar wavefront and curved wavefront. Moreover, the optimal structure of subarrays and subarray selection criteria are further investigated to make good use of our approach in practical applications.

This paper contributes to the area of 3-D source localization in the following three aspects:

1 For the source’s 3-D localization, this paper provides a novel ambiguity resolution method to obtain the source’s actual parameters with satisfactory performance and low computational complexity.
2 As the proposed algorithm does not need rotation, it can achieve real-time source localization, thus the applied scope is wider than the rotary way to resolve ambiguity.
3 The optimal structure of subarrays and subarray selection criteria can be exploited in SGAS to improve the performance and practicability of our approach.

The rest of the paper consists of seven sections. Section 2 reviews the model of a narrowband source’s 3-D parameter estimation. Section 3 presents the source’s angle estimation under an unambiguous situation and the method of SGAS. Section 4 introduces the method of phase-based parameter estimation on the basis of SGAS (SGAS-PBPE) to obtain the source’s range and more precise angle estimation. Section 5 discusses the optimum structure of subarray and subarray selection criteria in terms of computational cost and estimation performance. Section 6 carries out simulations to demonstrate the effectiveness of SGAS, theoretical analysis of optimum subarray selection, and to compare estimation performance between SGAS and SGAS-PBPE. Section 7 concludes the whole paper.

## 2. Signal Modeling

**Figure 1:**
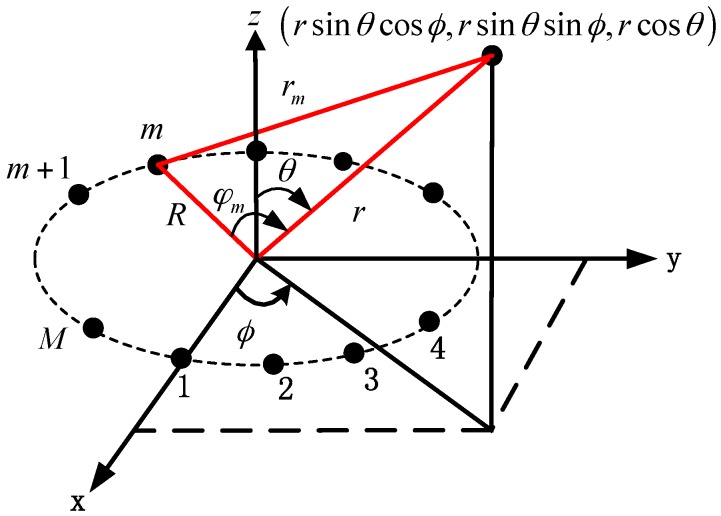
Signal model.

Consider a narrowband radiating source impinging on a fixed UCA with radius *R* and *M* identical omni-directional sensors uniformly spaced on the circumference in the *xy*-plane, where *M* is an even integer. The geometry of the signal model is shown in Figure 1. The source is located at (ϕ,θ,r), where the azimuth angle ϕ∈[−π,π) is measured counterclockwise from the *x*-axis, the elevation angle θ∈[0,π/2) is measured downward from the *z*-axis, and the range r is measured from the center of the UCA.

Here, we assume that the range of the source with a curved wavefront is beyond the Fresnel area, but dissatisfies the condition of far-field. Under this assumption, the observation vector of the mth sensor at time n can be expressed as

(1) $$x_{m}(n)=s\left(n\right)\exp\left\{j\frac{2\pi }{\lambda }\left(r-r_{m}\left(\phi ,\theta ,r\right)\right)\right\}+w_{m}(n)$$

for m=1,…,M, n=1,…,N. s(n) is assumed to be a zero-mean complex envelope with power $\sigma_{s}^{2}$, and $w_{m}(n)$ is assumed to be a zero-mean white complex Gaussian noise with power $\sigma_{n}^{2}$, which is independent of s(n). λ is the wavelength of the source. $r_{m}(\phi ,\theta ,r)$ is the range between the *m*th sensor and the source, which is given by

(2) $$r_{m}(\phi ,\theta ,r)=r\sqrt{1+{(R/r)}^{2}-2R\zeta_{m}(\phi ,\theta )/r}$$

where $\zeta_{m}(\phi ,\theta )=\cos\varphi_{m}=\cos(\gamma_{m}-\phi )\sin\theta$ with $\gamma_{m}=2\pi (m-1)/M$, and the angle $\varphi_{m}$ is measured counterclockwise from the line of centre of UCA and the mth sensor. Consider a function defined as

(3) $$f(\iota_{r})={\left\{1+\iota_{r}^{2}-2\iota_{r}\cdot \zeta_{m}(\phi ,\theta )\right\}}^{1/2}$$

where $\iota_{r}=R/r$. According to a sufficiently large value r relative to R, $f(\iota_{r})$ can be well approximated by a second-order Taylor series expansion as

(4) $$f(\iota_{r})\approx f(0)+f^{\prime }(0)\iota_{r}+\frac{1}{2}f^{\prime \prime }(0)\iota_{r}^{2}$$

Then, $r_{m}(\phi ,\theta ,r)$ can be approximated as 

(5) $$r_{m}(\phi ,\theta ,r)\approx r-R\zeta_{m}(\phi ,\theta )+\frac{R^{2}}{2r}\left(1-\zeta_{m}^{2}(\phi ,\theta )\right)$$

Substituting (2) and (5) into (1) yields the approximated expression of observation vector as

(6) $$x_{m}(n)=s(n)\exp\left\{j\frac{2\pi R}{\lambda }\left(\zeta_{m}(\phi ,\theta )-\frac{R}{2r}\left(1-\zeta_{m}^{2}(\phi ,\theta )\right)\right)\right\}+w_{m}(n)$$

It can be seen from (6) that the source’s 3-D parameters are included in its exponent, so we intend to estimate them by utilizing the phase of receiving data.

## 3. Ambiguity Resolution of Source’s Angles

As we know, the ambiguity of 3-D parameter estimation mainly focuses on 2-D angle estimation. However, ambiguity resolution becomes quite difficult as the source’s angles interweave with range. Thus, we first tend to decouple the source’s angles and range via the centro-symmetric configuration of UCA. By utilizing some algebraic operations, the source’s angles are then obtained under an unambiguous situation. Finally, considering an ambiguous situation, subarray grouping and ambiguity searching are employed to obtain actual angle parameters.

### 3.1. Source’s Angle Estimation under Unambiguous Situation

Note that ambiguity in parameter estimation is mainly induced by angle parameters. The range parameter is considered as a nuisance, which causes difficulty in ambiguity resolution. Hence, we should take some measures to separate the source’s angles and range.

According to the array configuration of UCA with even number sensors, it can be noticed that $\gamma_{m+M/2}=\gamma_{m}+\pi$, and then

(7) $$\zeta_{m+M/2}(\phi ,\theta )=-\zeta_{m}(\phi ,\theta )$$

for m=1,2,…,M/2. By using the property in (7), consider a correlation function defined as

(8) $$R_{m}=E\left\{x_{m}(n)x_{m+M/2}^{*}(n)\right\}\;=\sigma_{s}^{2}\exp\left\{j\frac{4\pi R}{\lambda }\zeta_{m}(\phi ,\theta )\right\}+\sigma_{n}^{2}\;$$

where ${\left(\cdot \right)}^{*}$ denotes the complex conjugate. Assuming that the receiving data is noiseless, the phase of $R_{m}$ is

(9) $$u_{m}=\arg(R_{m})=4\pi R\zeta_{m}(\phi ,\theta )/\lambda +2\pi q$$

where q is a certain integer and the range parameter is counteracted.

**Figure 2:**
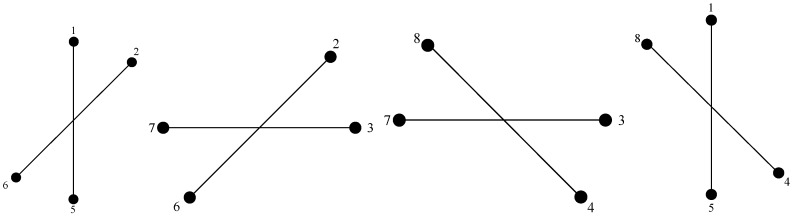
Subarray grouping with M=8.

Firstly, we consider the unambiguous situation q=0 and employ two couples of adjacent CCS to form subarray for deducing easily. Taking a UCA with M=8 as an example, subarrays after grouping are shown in Figure 2. Based on trigonometric function, we can simplify the sum and the difference between $u_{m}$ and $u_{\mathrm{mod}(m+1,M/2)}$, which can be written as

(10) $$v_{m}=u_{m}+u_{\varepsilon_{m,1}}\;=\frac{8\pi R}{\lambda }\sin(\theta )\cos(\rho \tau_{m,1}-\phi )\cos(\rho \upsilon_{m,1})$$

(11) $$w_{m}=u_{m}-u_{\varepsilon_{m,1}}=-\frac{8\pi R}{\lambda }\sin(\theta )\sin(\rho \tau_{m,1}-\phi )\sin(\rho \upsilon_{m,1})$$

where $\varepsilon_{m,1}=\mathrm{mod}(m+1,M/2)$, ρ=π/M, $\tau_{m,1}=m+\varepsilon_{m,1}-2$, $\upsilon_{m,1}=m-\varepsilon_{m,1}$.

Then, by utilizing (10) and (11), a plural can be constructed as

(12) $$z_{m}=\frac{v_{m}}{8\pi R/\lambda \cdot \cos(\rho \upsilon_{m,1})}-j\frac{w_{m}}{8\pi R/\lambda \cdot \sin(\rho \upsilon_{m,1})}=\exp\left(j(\rho \tau_{m,1}-\phi )\right)\sin(\theta )$$

Further, by eliminating the known parts, the plural which only includes source’s angles can be expressed as

(13) $$z^{\prime }=z_{m}^{*}\exp\left(j\rho \tau_{m,1}\right)=\exp(j\phi )\sin(\theta )$$

Finally, the expression of source’s angles is given by

(14) $$\phi =\mathrm{angle}(z^{\prime })$$

(15) $$\theta =\arcsin\left(\left|z^{\prime }\right|\right)$$

Under the unambiguous assumption, the actual source’s angles can be obtained via the above procedure. In practice, ambiguity always appears in the source’s localization and it is quite significant to investigate effective schemes of ambiguity resolution. Moreover, it should be pointed out that using two couples of adjacent CCS to form a subarray is only considered for ease of deduction, and the different structures of subarray will be discussed in the complementary section.

### 3.2. Ambiguity Resolution by Using the Method of SGAS

To guarantee no phase ambiguity in (9), the condition R≤λ/4 is commonly demanded, but when the array has been designed, it would be always invalid, especially for localization of a high-frequency source. For fixed UCA, as each subarray can obtain the actual source’s angles under a certain ambiguity, we employ traverse of ambiguity to make each subarray acquire a batch of results and search the nearest value among subarrays to ascertain actual source’s angles.

Firstly, the maximum ambiguity of phase difference $u_{m}$ can be computed by

(16) D=ceil(2R/λ)

where ceil(⋅) denotes upper nearest integer. Then, based on traverse of ambiguity, the phase difference matrix U is expressed as

(17) $$U=\left[\begin{array}{cccc} u_{1}-2\pi D & u_{2}-2\pi D & \cdots & u_{M/2}-2\pi D\\ u_{1}-2\pi (D-1) & u_{2}-2\pi (D-1) & \cdots & u_{M/2}-2\pi (D-1)\\ \vdots & \vdots & \cdots & \vdots \\ u_{1}+2\pi D & u_{2}+2\pi D & \cdots & u_{M/2}+2\pi D \end{array}\right]$$

**Figure 3:**
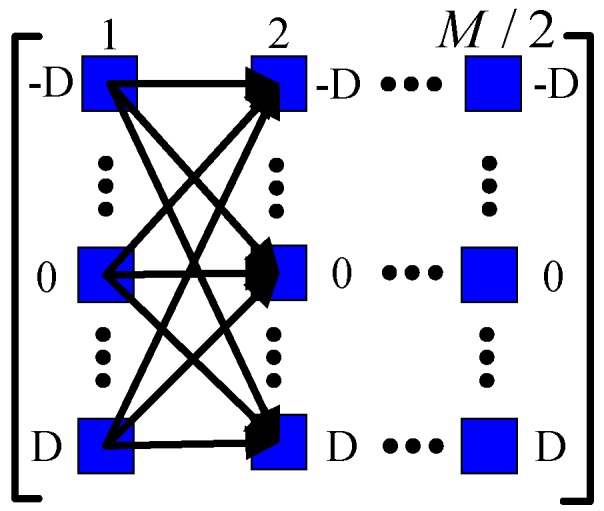
The sketch map of the plural matrix (PM) computation process.

Finally, similar to (10)–(13), any two phase differences of adjacent columns in (17) could develop a plural, and there are ${\left(2D+1\right)}^{2}$ combinations. Thus, we can obtain a plural matrix (PM), which is given by

(18) $$Z=\left[\begin{array}{cccc} z_{1,\;1}^{\prime } & z_{2,\;1}^{\prime } & \cdots & z_{M/2,\;1}^{\prime }\\ z_{1,\;2}^{\prime } & z_{2,\;2}^{\prime } & \cdots & z_{M/2,\;2}^{\prime }\\ \vdots & \vdots & \vdots & \vdots \\ z_{1,\;\mu^{2}}^{\prime } & z_{2,\;\mu^{2}}^{\prime } & \cdots & z_{M/2,\;\mu^{2}}^{\prime } \end{array}\right]$$

where μ=2D+1, and $z_{\varpi ,\delta }^{\prime }$ in (18) for ϖ=1,2,...,M/2, $\delta =1,2,...,\mu^{2}$ is computed by the ξth element of the ϖth column and the ϑth element of the ςth column in (17), where ξ=ceil(δ/μ), ϑ=mod(δ/μ), ς=mod((ϖ+1)，M/2). The sketch map of computation process is shown in Figure 3. 

It is worth noting that the value of each column in PM represents a batch of subarray results under different ambiguities and each column only includes a plural constructed by the actual source’s angles. Therefore, the source’s unambiguous angles can be obtained by finding the nearest value among subarrays.

Firstly, we take the first column in PM as the reference and select an element from the referenced column in turn. Then, the sum of minimum difference between the element of referenced column and the element of other columns can be given by

(19) $$d_{p}=\sum_{k=2}^{M/2}\left(\underset{h=1,2,...,\mu^{2}}{\min}\left|z_{1,p}^{\prime }-z_{k,h}^{\prime }\right|\right)$$

for $p=1,2,...,\mu^{2}$. It is clear that the minimum sum of differences among columns is brought from actual angles. Finally, by finding the coordinate of minimum value in (19) and its corresponding plural, the source’s unambiguous angles can be obtained by

(20) $$g=\underset{p}{\arg\min}\left(d_{p}\right)$$

(21) $${\hat{\phi }}^{\prime }=\mathrm{angle}(z_{1,g}^{\prime })$$

(22) $${\hat{\theta }}^{\prime }=\arcsin\left(\left|z_{1,g}^{\prime }\right|\right)$$

In practical application, the phase difference $u_{m}$ would be replaced with its estimate ${\hat{u}}_{m}=\arg({\hat{R}}_{m})$, where a finite sample estimate ${\hat{R}}_{m}=(1/N)\sum_{n=1}^{N}x_{m}(n)x_{m+M/2}^{*}(n)$. In addition, considering computational cost, not all the columns in PM are necessary to find the coordinate in (20), and the detailed research will be presented in a subsequent section.

The procedure of SGAS could be summarized as

Step 1 To ascertain the scope of ambiguity, the maximum ambiguity of phase difference D needs to be calculated firstly according to the equation in (16).
Step 2 After subarray grouping, the phase difference estimate ${\hat{u}}_{m}$ can be computed by using receiving data of each subarray. To find the unambiguous phase difference, phase difference matrix $\hat{U}$ including actual phase difference can be obtained by utilizing the traverse of ambiguity.
Step 3 As the unambiguous phase differences of different subarrays could form the same plural, the plural matrix $\hat{Z}$ is computed based on $\hat{U}$ and the Equations (10)–(13) to estimate source’s angles without ambiguity.
Step 4 As each column of matrix $\hat{Z}$ includes plural value constructed by the actual angles of the source, the method of searching the most similar plural matrix terms by minimizing magnitude of difference in the Equations (19) and (20) can be utilized to obtain the coordinate corresponding to unambiguous angles. The ambiguity-free estimation of the source’s angles $\left({\hat{\phi }}^{\prime },{\hat{\theta }}^{\prime }\right)$ are then computed by substituting the coordinate into Equations (21) and (22).

## 4. 3-D Parameter Estimation of Source

It can be noticed that each subarray only utilizes four sensors to obtain the aforementioned angle estimation. According to the analysis of Cramer Rao bound (CRB) in [17], the estimation performance improves as the number of sensors increases, so we can employ more sensors to make the source’s angle estimation more precise. In terms of computational complexity and estimation performance, the phase-based algorithm in [11] has obvious advantages. Considering that the steering vectors of sources with planar wavefronts and curved wavefronts could be well approximated as a sufficiently large value *r* relative to *R*, we utilize the unambiguous angle estimation of SGAS to resolve the ambiguity in phase-based 3-D parameter estimation algorithm called SGAS-PBPE, which could acquire the source’s range and more precise angles.

Sufficient theoretical results and simulative/experimental ones demonstrate that, the longer the baseline is, the higher the DOA estimation precision will be [15]. The longest baseline is just constructed by CSS, but the source’s range parameter cannot be estimated. Therefore, we select the sub-longest baseline to obtain the source’s 3-D parameter estimation.

According to (8) and (9), the phase difference using the sub-longest baseline can be expressed as

(23) $$u_{m}^{\prime }=\frac{2\pi R}{\lambda }\left\{\psi_{m}(\phi ,\theta ,r)-\psi_{m^{\prime }}(\phi ,\theta ,r)\right\}+2\pi \kappa$$

for m=1,2,…,M, where $m^{\prime }=\mathrm{mod}(m+M/2-1,M)$, $\psi_{m}(\phi ,\theta ,r)=\zeta_{m}(\phi ,\theta )-\frac{R}{2r}\left(1-\zeta_{m}{\left(\phi ,\theta \right)}^{2}\right)$ and κ is a certain integer. Similarly, the phase difference $u_{m}^{\prime }$ would be replaced with its estimate ${\hat{u}}_{m}^{\prime }=\arg({\hat{R}}_{m}^{\prime })$, where a finite sample estimate ${\hat{R}}_{m}^{\prime }=(1/N)\sum_{n=1}^{N}x_{m}(n)x_{m^{\prime }}^{*}(n)$.

It is clear that the steering vector of a source with planar wavefront lacks the section $-2\pi R^{2}\left(1-\zeta_{m}{\left(\phi ,\theta \right)}^{2}\right)/\lambda r$ compared with the steering vector of a source with curved wavefront. Yet, according to a sufficiently large value r relative to R, these two steering vectors can be well approximated. Thus, the unambiguous phase difference in (23) can be estimated by using the actual angle estimation obtained by SGAS, which is given by

(24) $$u_{m}^{\prime \prime }=\frac{2\pi R}{\lambda }\left\{\zeta_{m}({\hat{\phi }}^{\prime },{\hat{\theta }}^{\prime })-\zeta_{m}({\hat{\phi }}^{\prime },{\hat{\theta }}^{\prime })\right\}$$

Based on (24), we can compute the ambiguity of phase difference in (23) and more precise estimation of unambiguous phase difference can be written as

(25) $${\hat{u}}_{m}^{\prime \prime \prime }={\hat{u}}_{m}^{\prime }-2\pi \kappa ={\hat{u}}_{m}^{\prime }-2\pi \cdot \mathrm{round}\left(({\hat{u}}_{m}^{\prime }-u_{m}^{\prime \prime })/2\pi \right)\;=\frac{2\pi R}{\lambda }\left\{\psi_{m}(\phi ,\theta ,r)-\psi_{m^{\prime }}(\phi ,\theta ,r)\right\}$$

where round(⋅) denotes rounding off. By observing (25) and referring [11], ${\hat{u}}_{m}^{\prime \prime \prime }$ can be reformulated as the form of matrix ${\hat{U}}^{\prime }=Ab$, where ${\hat{U}}^{\prime }={\left[\begin{array}{cccc} {\hat{u}}_{1}^{\prime \prime \prime } & {\hat{u}}_{2}^{\prime \prime \prime } & \ldots & {\hat{u}}_{M}^{\prime \prime \prime } \end{array}\right]}^{T}$, matrix $A=[a_{m,1},a_{m,2},a_{m,3},a_{m,4}]$ and

(26) $$b=\frac{2\pi R}{\lambda }\left[\begin{array}{c} \cos(\phi )\sin(\theta )\\ \sin(\phi )\sin(\theta )\\ \left(\frac{R}{4r})\cos(2\phi )\sin^{2}(\theta \right)\\ \left(\frac{R}{4r})\sin(2\phi )\sin^{2}(\theta \right) \end{array}\right]$$

where $a_{m,1}=\cos(\gamma_{m}-\gamma_{m^{\prime }})$, $a_{m,2}=\sin(\gamma_{m}-\gamma_{m^{\prime }})$, $a_{m,3}=\cos(2\gamma_{m}-2\gamma_{m^{\prime }})$, and $a_{m,4}=\sin(2\gamma_{m}-2\gamma_{m^{\prime }})$.

Notice that only the matrix b includes the 3-D parameters of the source, so the optimal solution b can be estimated by employing the least square (LS) $\hat{b}={\left[\begin{array}{cccc} {\hat{b}}_{1} & {\hat{b}}_{2} & {\hat{b}}_{3} & {\hat{b}}_{4} \end{array}\right]}^{T}={\left(A^{T}A\right)}^{-1}A^{T}{\hat{U}}^{\prime }$. As a result, from (26), the estimates of (ϕ,θ,r) can be obtained.

## 5. Complementary Issues to SGAS

In this section, to make good use of the proposed ambiguity resolution algorithm SGAS in practical application, we will discuss some complementary issues to SGAS, including the optimum structure of the subarray and subarray selection criteria.

### 5.1. Selection of Subarray Structure

**Figure 4:**
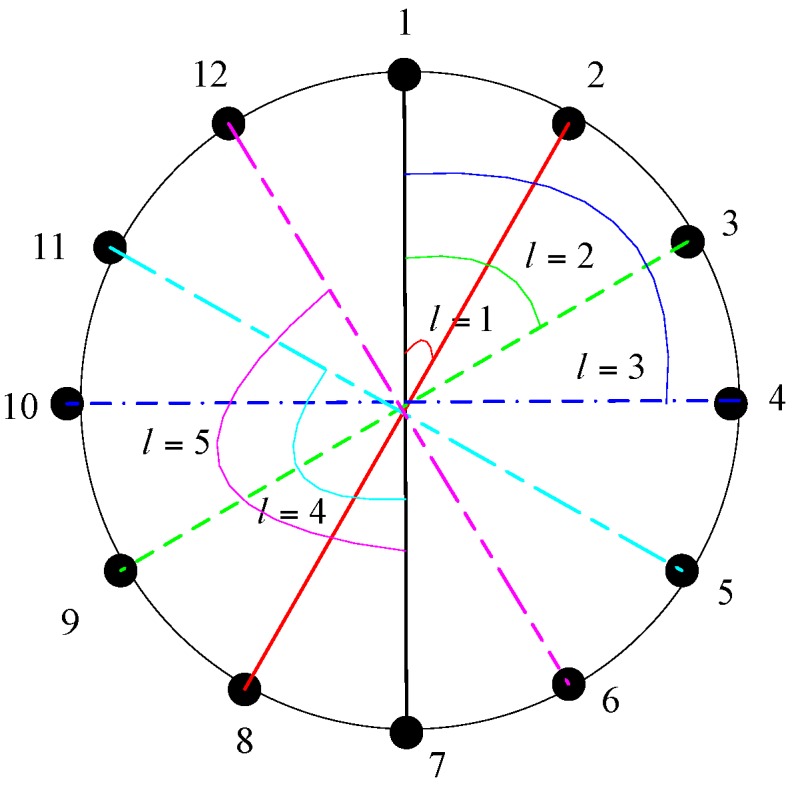
Different structure of a subarray versus *l*.

In the algorithm procedure above, the source’s angles are deduced via a subarray formed by two couples of adjacent CSS. In fact, a subarray formed by two different CSS groups could obtain the estimation of the source’s angles. The formula in (10) and (11) could be rewritten as

(27) $$v_{m}^{\prime }=u_{m}+u_{\varepsilon_{m,l}}\;=\frac{8\pi R}{\lambda }\sin(\theta )\cos\left(\rho (\tau_{m,l}-\phi )\right)\cos(\rho \upsilon_{m,l})$$

(28) $$w_{m}^{\prime }=u_{m}-u_{\mathrm{mod}(m+l,M/2)}=-\frac{8\pi R}{\lambda }\sin(\theta )\sin\left(\rho (\tau_{m,l}-\phi )\right)\sin(\rho \upsilon_{m,l})$$

where l=1,2,…,M/2−1, which represents the space of two couples of CSS, $\varepsilon_{m,l}=\mathrm{mod}(m+l,M/2)$, $\tau_{m,l}=m+\varepsilon_{m,l}-2$, $\upsilon_{m,l}=m-\varepsilon_{m,l}$. As the subsequent procedure of the source’s angle parameter estimation is similar to (12)–(15), the details are not provided here. By taking phase difference $u_{1}$ as the reference, the different structures of subarray with M=12 versus l is shown in Figure 4.

**Figure 5:**
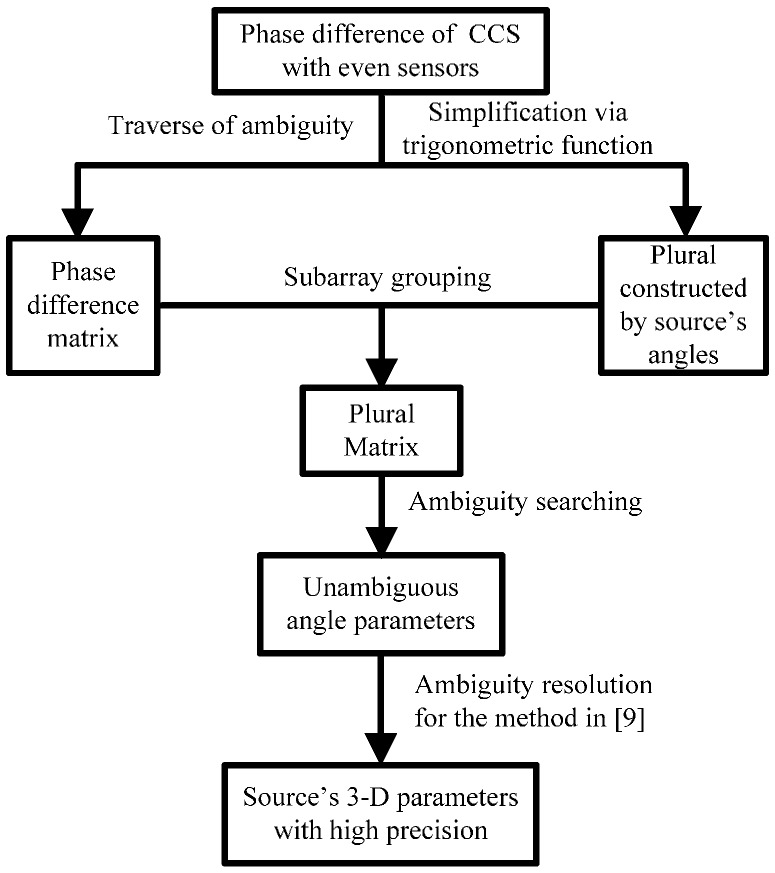
Flow chart of the proposed algorithm SGAS-PBPE.

In addition, it is clear that the estimation performance of the source’s angle parameter is not the same for different l. In fact, the selection of the optimal subarray structure, which provides the best estimation performance, is a problem in the performance comparison of array geometry. In Section 1 we have pointed out several advantages of UCA over the other geometries, thus the best estimation performance should be brought by the selection of a subarray whose structure is similar to a UCA with four sensors. Taking M=12 in Figure 5 as an example, the best selection might be two couples of CSS with l=3. Meanwhile, we will demonstrate the conclusion in the subsequent simulation section.

### 5.2. Criteria of Subarray Selection

In the practical application of SGAS, it is unnecessary to employ all results of subarrays under different ambiguities, which would cause high computational complexity. Therefore, it is significant to research proper subarray selection in terms of computational cost and effectiveness of ambiguity resolution.

A batch of results corresponds to a column in PM. It is clear that two columns in PM are the least which we can employ. Yet, how to properly select two columns in PM to resolve ambiguity effectively needs to be further investigated.

First, we intend to analyze the selection of columns by employing the structure of a subarray formed by two couples of adjacent CSS. In an ambiguous situation, the sum and difference $u_{m}$ and $u_{\varepsilon_{m,1}}$ in (10) and (11) can be expressed as

(29) $$v_{m}^{\prime \prime }=v_{m}+2\pi \alpha_{m}$$

(30) $$w_{m}^{\prime \prime }=w_{m}+2\pi \beta_{m}$$

where $-2D\leq \alpha_{m},\beta_{m}\leq 2D$, and they are certain integer, representing the ambiguities of sum and difference of two CCS groups, respectively. Based on (12) and (13), the new plural can be written as

(31) $$z_{m}^{\prime }=z^{\prime }+\frac{\lambda }{4R}\left(\frac{\alpha_{m}}{\cos(\rho \upsilon_{m,1})}+j\frac{\beta_{m}}{\sin(\rho \upsilon_{m,1})}\right)\exp(j\rho \tau_{m,1})$$

When $\left|\alpha_{m}\right|\neq \left|\beta_{m}\right|$, the plural value among columns would be equivalent only under the actual source’s angles. However, when the ambiguities of one subarray satisfy $\alpha_{\chi }=\beta_{\chi }$, (31) can be simplified as

(32) $$z_{\chi }^{\prime }=z^{\prime }+j\frac{\lambda \alpha_{\chi }\exp\left(j\rho (\tau_{\chi ,1}-\upsilon_{\chi ,1})\right)}{4R\sin(\rho \upsilon_{\chi ,1})\cos(\rho \upsilon_{\chi ,1})}=z^{\prime }+j\frac{\lambda \alpha_{\chi }\exp\left\{2j\rho \left(\mathrm{mod}(\chi +1,M/2)-1\right)\right\}}{4R\sin(\rho \upsilon_{\chi ,1})\cos(\rho \upsilon_{\chi ,1})}$$

And at the same time, if the ambiguities of the adjacent subarray satisfy $\alpha_{\chi +1}=-\beta_{\chi +1}$, (31) can be simplified as

(33) $$z_{\chi +1}^{\prime }=z^{\prime }+j\frac{\lambda \alpha_{\chi +1}\exp\left(j\rho (\tau_{\chi +1,1}+\upsilon_{\chi +1,1})\right)}{4R\sin(\rho \upsilon_{\chi +1,1})\cos(\rho \upsilon_{\chi +1,1})}=z^{\prime }+j\frac{\lambda \alpha_{\chi +1}\exp\left(2j\rho \chi \right)}{4R\sin(\rho \upsilon_{\chi +1,1})\cos(\rho \upsilon_{\chi +1,1})}$$

It should be noted that $\upsilon_{\chi +1,1}=\upsilon_{\chi ,1}$ and χ=mod(χ+1,M/2)−1 for χ=1,2,…,M/2−1. Satisfying the conditions in (32)–(33), the plural value of adjacent columns would be equivalent, which causes the ineffectiveness of ambiguity resolution for multiple nearest value.

Moreover, for a different structure of subarrays, ambiguity resolution would be ineffective under similar conditions. For example, we only employ the first and the third columns in PM to resolve ambiguity with M=12 and l=2. Furthermore, we find that the value of ineffective combinations are computed by four CSS groups with a common group. In terms of the aforementioned example, the results in the two ineffective columns are obtained based on a common CSS group constructed by 3th sensor and 9th sensor respectively. 

Therefore, we would reach the significant conclusion that the SGAS algorithm needs at least four couples of different CSS, thus, the number of sensors should be no less than eight to guarantee the effectiveness of ambiguity resolution.

## 6. Experiments

In this section, we carry out simulations to demonstrate the satisfying performance of our proposed algorithm and support the theoretical results. A UCA with M=12 and fixed array radius R=0.5 m is employed. Three hundred Monte Carlo runs are performed to obtain the probability of precise estimation and the root mean square errors (RMSEs) of the source’s parameter estimation.

Simulation experiments are divided into four parts. In Part A, the effectiveness of SGAS is firstly presented. Then, by defining precise estimation as the difference between parameter estimation and actual value being no more than one degree, the probability of precise estimation $P_{pe}=(N_{pe}/N_{mo})\cdot 100\%$ representing the ratio between the count of precise estimation *N_pe_* and Monte Carlo runs *N_mo_* is utilized to illustrate the applied scope of SGAS. In Part B, we mainly research the optimal subarray structure by employing the RMSEs of the source’s angle parameter estimation versus azimuth angle under different structures of subarray. In Part C, considering subarray selection criteria, the computational time of different numbers of subarray versus the source’s frequency is firstly compared, and the effectiveness of the source’s angle estimation under different combinations of two subarrays is then shown to validate theoretic analysis in part B of Section 4. Finally, the comparison of estimation performance between SGAS and SGAS-PBPE is given in Part D. It should be pointed out that by using the SGAS method, the unambiguous source’s angles are utilized to estimate the source’s range via the 1-D MUSIC algorithm, which compares with range estimation using the method of SGAS-PBPE.

### 6.1. Effectiveness of SGAS

**Figure 6:**
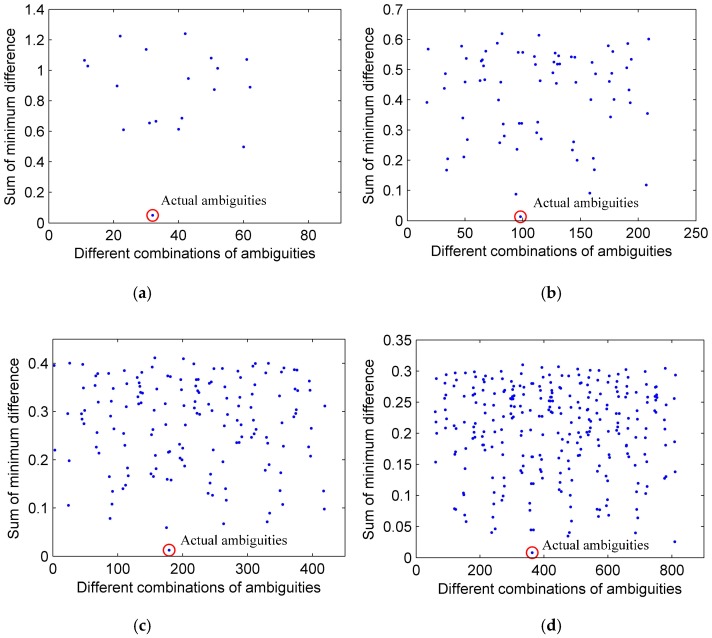
Sum of minimum difference versus different combinations of ambiguities under different source frequencies. (**a**) f=1 GHz; (**b**) f=2 GHz; (**c**) f=3 GHz; (**d**) f=4 GHz.

Suppose that the source is located at $\left(\phi ,\theta ,r\right)=\left(120.5^{\circ },\;20.1^{\circ },\;6.5\;m\right)$, and the signal-to-noise ratio (SNR) is 10 dB. Each subarray is formed by two couples of adjacent CSS and all subarrays are employed in ambiguity searching. As the scope is wide, the sum of minimum difference between the element of the column referenced and the element of other columns in (19) is partially shown and four typical source frequencies f=1 GHz, f=2 GHz, f=3 GHz, f=4 GHz are considered in Figure 6. It can be seen that the blue points represent the sum of minimum difference under different combinations of ambiguities corresponding to different values of *p* in (19), and the red circle represents the actual ambiguities corresponding to the minimum value of the sum in (19), which illustrates the effectiveness of SGAS. In addition, it can also be noted that the number of ambiguity combinations increases as the source’s frequency rises, and the difference between minimum value and other value reduces as the source’s frequency rises.

**Figure 7:**
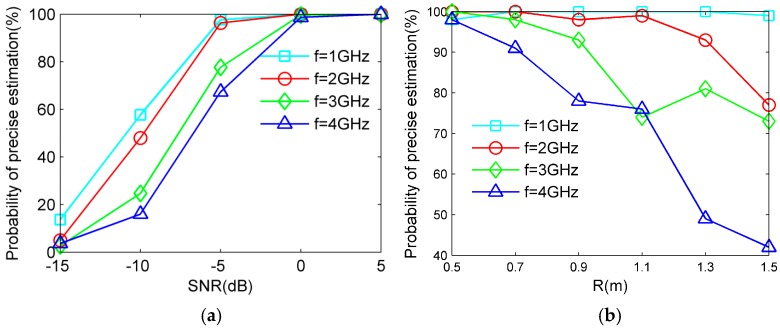
Probability of precise estimation under different source’s frequencies. (**a**) variational SNR; (**b**) variational radius of uniform circular array (UCA).

Moreover, to present the applied scope of SGAS, under the corresponding frequencies of source in Figure 6, the probabilities of precise source angle estimation versus SNR and the radius of UCA are given in Figure 7, respectively. As shown in Figure 7a, the probabilities of precise estimation rise as the SNR increases, and all the probabilities under different source frequencies are acceptable when SNR is more than 0 dB. Meanwhile, when compared with a low frequency of source, the probability of precise estimation for a high-frequency source is smaller, especially under low SNR, since the superiority of minimum value reduces as the source’s frequency rises (shown in Figure 6), which causes the ineffectiveness of SGAS more easily for a high-frequency source at low SNR. As shown in Figure 7b, the probabilities of precise estimation decrease as the radius of UCA increases, and all the probabilities under different source’s frequencies are acceptable when the radius is less than 0.7 m. Similarly, it is easier for a high-frequency source to cause the ineffectiveness of the SGAS algorithm under a large radius of UCA. Consequently, the proposed SGAS algorithm is effective and the applied scope including frequency of ambiguity resolution, acceptable SNR and the maximum aperture is comparatively wide.

### 6.2. The Optimal Structure of Subarray

In this part, we will demonstrate the theoretical analysis in part A of Section 4 by simulation experiments, and the conclusion that the optimal structure of subarray could be reached.

**Figure 8:**
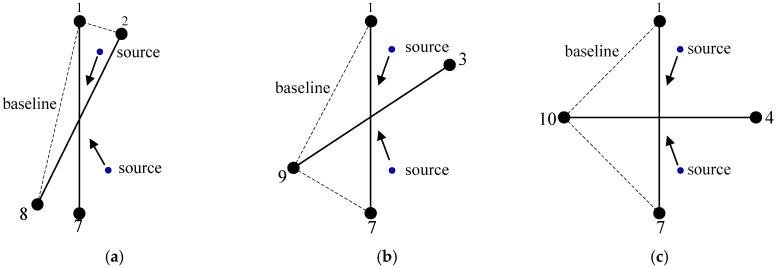
Sketch map of source’s azimuth angle estimation under three structures of subarray with M=12. (**a**) l=1; (**b**) l=2; (**c**) l=3.

**Figure 9:**
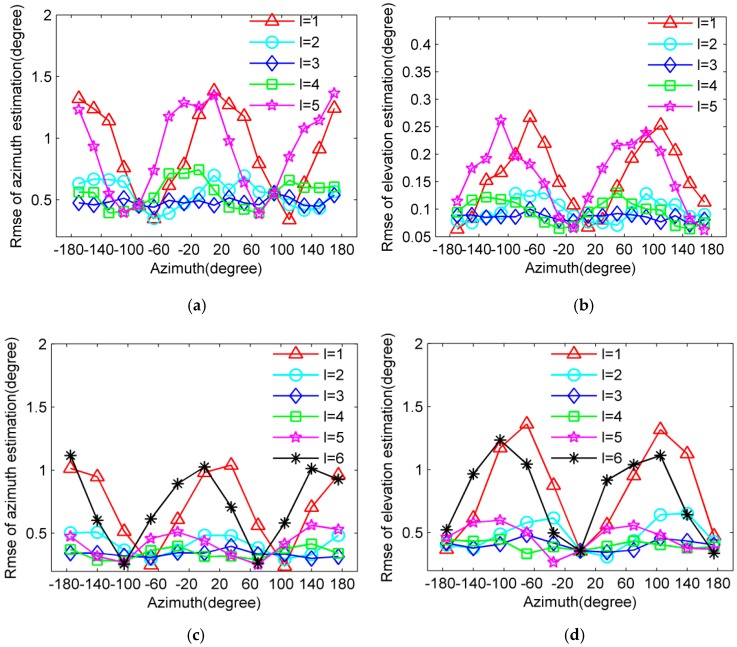
Root mean square errors (RMSEs) of the source’s angle parameters versus azimuth angle. (**a**) Azimuth angle (M=12); (**b**) Elevation angle (M=12); (**c**) Azimuth angle (M=14); (**d**) Elevation angle (M=14).

We take three structures of subarray with M=12 as an example. It can be seen in Figure 8 that for the different source’s azimuth angle, estimation using different lengths of baseline would cause distinguishing performance, so the source’s azimuth angle estimation is sensitive to the structure of subarray. Therefore, to search the optimal structure of subarray, the RMSEs of the source’s angle parameter estimation versus azimuth angle under different structures of subarray are investigated, which are given in Figure 9. Suppose that the unambiguous frequency of source is f=100 MHz, the fixed source’s elevation angle is $\theta =50^{\circ }$, the source’s azimuth angle varies from $-180^{\circ }$ to $180^{\circ }$, and all the subarrays are utilized. It can be found that the optional structure of subarray corresponding to CCS space for M=12 is l=1, 2, 3, 4, 5 and for M=14 is l=1, 2, 3, 4, 5, 6. The subarray structure with M=12 and space l=3 is similar to a UCA with four sensors and the subarray structure with M=14 and space l=3, 4 are both similar to a UCA with four sensors. Note that both of the changes of RMSEs with l=3 versus azimuth angle in Figure 9a,b are the slightest, and the estimation performance of the source’s angles is satisfactory. Similarly, the change of RMSEs with *l* = 3, 4 versus azimuth angle in Figure 9c,d are almost slightest, and the estimation performance of the source’s angles is also satisfactory. Thus, the simulation results indicate that the conclusion in part A of Section 4 is correct. 

### 6.3. The Criteria of Subarray Selection

In this part, the complementary issues to SGAS in part B of Section 4 will be demonstrated in simulation experiments, and the conclusion that the criteria of subarray selection could be obtained is discussed.

**Figure 10:**
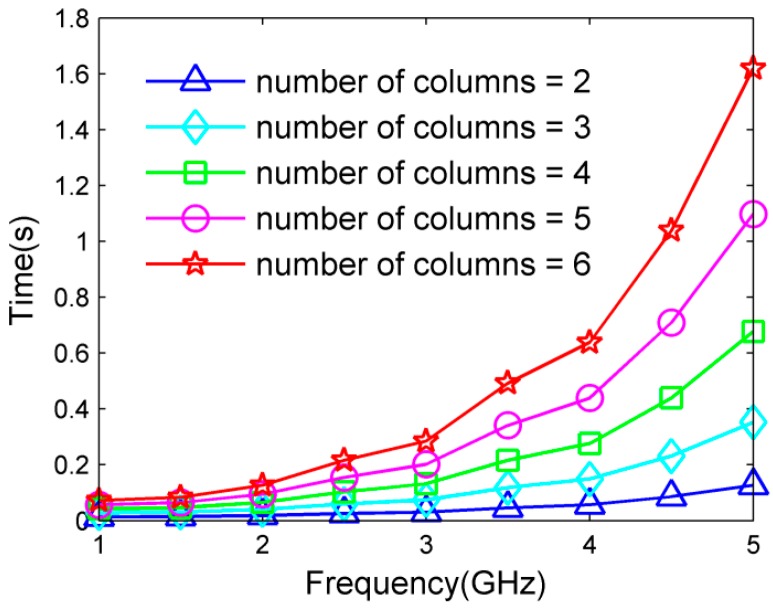
Computational time versus source’s frequency under different number of columns in PM.

For subarray selection criteria, the computational time using a different number of columns in PM versus source’s frequency is first compared. The results in Figure 10 indicate that the computational time increases as columns are added, especially for the angle parameter estimation of a high-frequency source. It is clear that employing only two columns in PM has advantages over other selections in terms of computational cost. Then, how to properly employ two columns for effective ambiguity resolution needs to be further researched.

**Figure 11:**
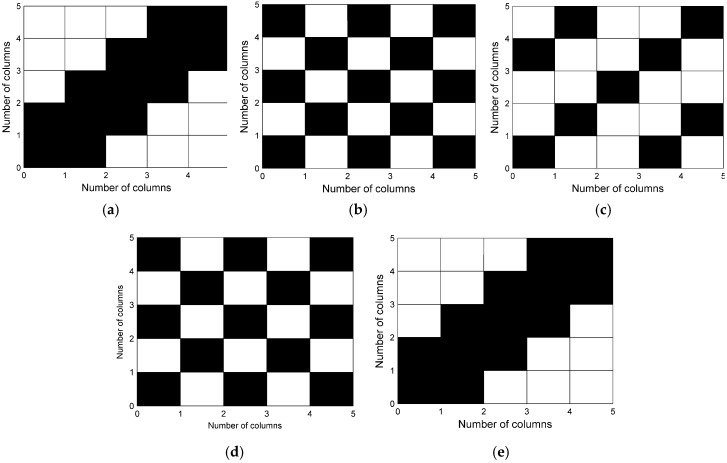
Effectiveness of precise estimation versus selection of columns in PM. (**a**) l=1; (**b**) l=2; (**c**) l=3; (**d**) l=4; (**e**) l=5.

Considering that only two columns are used in PM with M=12 and all space l of CSS groups are analyzed, the effectiveness of precise estimation with f=3 GHz under different combinations of two columns is shown in Figure 11. It can be seen from Figure 11 that the abscissa and ordinate of squares show the number of employed column combination in PM and the black squares represent the combinations which are ineffective on the precise estimation of the source’s angles, while the other white squares represent the effective combinations. In Figure 11a, under the space l=1 of CSS groups, it can be seen that the ambiguity resolution with the combination of same two columns or adjacent two columns in PM would be ineffective as SGAS has multiple coordinates of minimum value in (19). However, other combinations of two columns can obtain precise parameter estimation. Under the space l=2 and l=3, ambiguity resolution with the combinations of the same two columns or two columns whose space equal to l in Figure 11b,c are also ineffective for similar reasons, and other combinations could perform well. In addition, under the space l=4 and l=5, ambiguity resolution with the combinations of the same two columns or two columns whose space equal to 2 and 1, respectively, is ineffective, which is different from the above situations but corresponds to the theory in part B of Section 4. By observing, we can further find that all the ineffective combinations of two columns in the five situations are computed by four CSS groups with at least a common group.

As a result, it could be demonstrated that only two subarrays are enough to accomplish the source’s angle parameter estimation unambiguously, by avoiding the ineffective combinations.

It can be concluded that two subarrays whose structure is similar to a UCA with four sensors, formed by different CSS groups, would be the best selection in practical applications of SGAS. Therefore, the least number of sensors employed by the SGAS technology is eight.

### 6.4. Comparison of Source’s 3-D Parameter Estimation Performance between SGAS and SGAS-PBPE

In this part, we will carry out simulations by utilizing RMSEs to compare a source’s 3-D parameter estimation performance between SGAS and SGAS-PBPE. The other simulation conditions are similar to the experiment in part A except that the source’s frequency is set at f=3 GHz, and the SNR is varied from 0 dB to 20 dB. To examine the source’s range estimate performance of SGAS-PBPE, we compare it with a 1-D MUSIC estimator based on unambiguous source’s angle parameters obtained by SAGS. It should be pointed out that the search step size of the 1-D MUSIC estimator for the source’s range is Δr=0.01 m.

**Figure 12:**
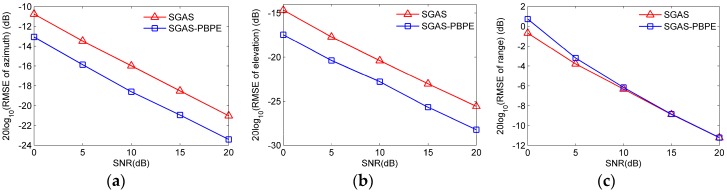
RMSEs of source’s 3-D parameter estimation versus SNR. (**a**) Azimuth angle; (**b**) Elevation angle; (**c**) Range.

The RMSEs of the source’s 3-D parameters are given in Figure 12a–c, respectively. As shown in Figure 12a,b, it is clear that the RMSEs of the source’s angle parameter estimation decrease monotonically with the SNR, and the SGAS-PBPE method provides improved estimation accuracy comparaed to the SGAS method. Note that Figure 12c, similar to source’s angle estimation, the RMSE of source’s range estimation also decline as SNR increases, and the estimation performance of SGAS-PBPE is analogous to the MUSIC estimator.

As a whole, simulation results indicate that the estimation performance of SGAS-PBPE outperforms the method of SGAS, which is consistent with the analysis given in Section 4.

## 7. Conclusions

In this paper, a novel ambiguity resolution algorithm is proposed for a source’s 3-D parameter estimation using UCA. Instead of collecting twice from different times, our approach utilizes subarray grouping and ambiguity searching by only one group of data, and so can adapt to fixed-frequency and frequency-hopping signals; which means it could be applied more broadly in practice than the rotary way to resolve ambiguity. Moreover, theoretical analysis and simulation experiments demonstrate the optimum structure of subarrays and subarray selection criteria, to make good use of the method. Simulation results show the effectiveness in ambiguity resolution and the satisfactory estimation performance of our approach. However, some drawbacks also exist in our proposed solution, including that the number of sensors is no less than eight and must be even.
